# Eumelanin from the skin of farm-raised giant salamanders promotes coagulation system homeostasis: a nutritional intervention for postoperative hemorrhage

**DOI:** 10.3389/fnut.2026.1896739

**Published:** 2026-08-18

**Authors:** Haobo Yuan, Yifei Zhang, Ruiqi Gong, Yuting Tan, Hao Han, Ching Yuan Hu

**Affiliations:** 1 Shaanxi Provincial Bioresource Key Laboratory, College of Biological Science and Engineering, Shaanxi Province Black Organic Food Engineering Technology Research Center, Shaanxi University of Technology, Hanzhong, Shaanxi, China; 2 School of Food Science and Technology, Shihezi University, Shihezi, Xinjiang, China; 3 Department of Human Nutrition, Food and Animal Sciences, College of Tropical Agriculture and Human Resources, University of Hawaii at Manoa, Honolulu, HI, United States

**Keywords:** *Andrias davidianus*, eumelanin, metabolomics, nutritional intervention, postoperative hemorrhage, UHPLC-Q-Orbitrap HRMS

## Abstract

**Background:**

Postoperative hemorrhage is a serious problem that impacts patient recovery. While current hemostatic agents are effective, they also pose a thrombosis risk. Therefore, it is critical to develop safe and natural nutritional intervention strategies.

**Objective:**

This study examines the regulatory effect of eumelanin from the skin of farm-raised giant salamanders (GS) on coagulation homeostasis and assesses its potential as a dietary supplement for postoperative recovery.

**Methods:**

The structure and biosafety of GS eumelanin were analyzed using SEM-EDS and FTIR, along with various other detection methods. Male Wistar rats (114) were used to evaluate coagulation function and for metabolomic analysis. We assessed tail bleeding parameters and coagulation indices at 2 and 24 h after treatment and analyzed intestinal metabolites 2 h after gavage.

**Results:**

SEM-EDS analysis reveals that the GS eumelanin has an irregular aggregated structure with Ca^2+^ involved in its formation. Multi-technique characterization (UV–Vis, FTIR, and XPS) confirmed the material as eumelanin, with characteristic optical absorption, surface functional groups, and elemental composition. Untargeted and targeted metabolomic analyses revealed that tranexamic acid and aminocaproic acid were detected in the intestinal metabolites of GS eumelanin. It also increases the levels of several other amino acids, including lysine, aspartic acid, and arginine, as well as the Ca^2+^ release. At 2 h post-gavage, the H-GS eumelanin group showed a 38.2% reduction in hemorrhage volume and a 27.0% reduction in hemostasis time compared with the CON group (*p* < 0.01). At the same time point, PT was 15.8 ± 1.0 s in H-GS eumelanin, compared to 17.3 ± 1.1 s in CON (*p* < 0.01). FIB was 230 ± 10 mg/dL in H-GS eumelanin versus 178 ± 7 mg/dL in CON (*p* < 0.001). Both values remained within the rat physiological ranges: 13.32–17.37 s for PT and 180–504 mg/dL for FIB. This indicates a favorable hemostatic safety profile, without any evidence of hypercoagulability.

**Conclusion:**

This study reveals that GS eumelanin regulates coagulation homeostasis through its nanostructure and intestinal metabolites. Our results provide a nutritional basis for using natural eumelanin as a functional food ingredient to help manage the risk of postoperative bleeding.

## 1 Introduction

Postoperative bleeding is a serious complication for hospital patients requiring surgery. A study of over 39,000 patients undergoing noncardiac surgery found that more than 11% experienced severe bleeding within the first 30 days after surgery, with 42.7% occurring within the first 24 h and 77.7% within the first week (1). Postoperative bleeding increases the financial burden on patients and severely affects their postoperative recovery (2, 3). Furthermore, anemia, bleeding, and the need for transfusions following noncardiac surgery have been linked to increased long-term mortality (4, 5).

Clinical management of postoperative bleeding typically involves the use of drugs to control bleeding and prevent complications such as hemorrhagic shock, organ dysfunction, infections, and delayed wound healing, which can significantly affect patient recovery and increase morbidity. Commonly used medications include tranexamic acid (TXA) and 6-aminocaproic acid (ACA). TXA has significantly reduced postoperative bleeding (6–8). However, careful dosing is essential, as excessive doses can increase the risk of thromboembolism by disrupting the fibrinolytic balance (9). In addition to medication, a balanced diet can help reduce postoperative bleeding. From a nutritional perspective, the postoperative metabolic state undergoes significant changes, including increased protein catabolism and greater substrate use for clotting factor synthesis. Therefore, nutritional support is an essential component of perioperative management.

The giant salamander (*Andrias davidianus*) is the largest extant amphibian in the world. It is prized for its tender and flavorful meat, which is considered highly nutritious (10–12). The giant salamander is primarily produced by captive breeding (13, 14). Additionally, the skin of the giant salamander has been utilized in traditional Chinese medicine. The eumelanin and other bioactive components in its skin are believed to promote wound healing and hemostasis (15).

Eumelanin is a type of biological macromolecule, with monomers including 5,6-dihydroxyindole (DHI) and 5,6-dihydroxyindole-2-carboxylic acid (DHICA) (16, 17). Natural eumelanin is commonly found in humans and animals. It is found in the tissues and organs of mammals, such as hair, skin, eyes, and feathers, as well as in the ink of cephalopods (18, 19). From a functional food perspective, eumelanin, like dietary polyphenols or polysaccharides, can be metabolized by the gut microbiota and enzymes into bioactive small molecules, exerting systemic nutritional regulation via a precursor-to-active-metabolite mode. While the various biological roles of eumelanin have been extensively studied, the hemostatic mechanism of eumelanin from the skin of the giant salamander has not been reported.

This study investigates the effect of eumelanin from the skin of farm-raised giant salamanders on systemic coagulation and its role as a dietary regulator in maintaining hemostatic balance. Although eumelanin is a well-known biomolecule, its physicochemical properties, including its morphological structure and metal ion composition, can vary significantly depending on the biological source. We selected eumelanin derived from the skin of farm-raised giant salamanders as the research subject because its morphological structure and biological activity have not been reported. Additionally, nanomaterials derived from natural sources are a popular research topic in medicine and food. This selection presents a significant opportunity to investigate its potential procoagulant mechanisms, which may be distinct from those of eumelanin derived from other origins. Previous research has primarily focused on the photoprotective and radical-scavenging properties of eumelanin (20), whereas the role of eumelanin and its intestinal metabolites in postoperative hemostasis remains largely unexplored. We demonstrate, for the first time, that eumelanin from the skin of giant salamanders and its key intestinal metabolites enhance coagulation. Additionally, we explain the metabolomic mechanisms behind this effect. These findings provide a strong, evidence-based foundation for developing targeted nutritional strategies to reduce the risk of postoperative bleeding during the perioperative period.

## 2 Materials and methods

### 2.1 Chemical reagents

Giant salamander skin was purchased from Hanzhong Longni Bioengineering Co., Ltd. (Hanzhong, China). Methanol, acetonitrile, and formic acid were UHPLC–MS grade and purchased from Thermo Fisher Scientific Inc. (Waltham, MA, USA). Tranexamic acid (TXA, CAS: 1197-18-8), 6-aminohexanoic acid (ACA, CAS: 60–32-2) (purity ≥98%), and alkaline protease (200 units/mg) standards were purchased from Shanghai Yuanye Biotechnology Co., Ltd. (Shanghai, China). A mixture of 20 standard amino acids was purchased from the National Metrological Research Institute. (Beijing, China). Calcium gluconate was purchased from Qingdao Nautical Science Co., Ltd. (Qingdao, China). PT kit, TT kit, APTT kit, FIB kit, and buffer were from Beijing Biochemistry Xinji Technology Development Co., Ltd. (Beijing, China).

### 2.2 Eumelanin extraction

We extracted eumelanin from the skin of Giant Salamanders using a modified method (21). First, we immersed the GS skin samples in a 6% NaCl solution for 60 min. Then, we treated the samples with 0.1% citric acid at 45 °C for 90 min in a water bath. After removing the mucus, we scraped the pigments, then subjected them to Soxhlet extraction with petroleum ether to remove the lipid fraction. After that, we applied a 5% papain solution and conducted enzymatic digestion at 55 °C for 4 h. Upon completing the enzymatic reaction, we collected the eumelanin-containing precipitate by centrifugation (2,683 g, 5 min). Then, the precipitate was washed with distilled water three times to remove residual enzymes and soluble proteins. We then mixed the treated precipitate with a 6% NaOH solution and centrifuged (2,683 g, 10 min) to separate the supernatant. We adjusted the supernatant pH to 1.0 using 6 mol/L hydrochloric acid to induce acid precipitation. We repeated the alkaline dissolution followed by acid precipitation process three times to increase the purity of the eumelanin. Finally, we thoroughly washed the precipitates with ethanol and distilled water and vacuum-dried them at 40 °C to obtain high-purity eumelanin from the Giant Salamanders (GS).

We assessed the extraction rate and purity of GS eumelanin. We calculated the extraction rate as a percentage. It was the mass of purified eumelanin divided by the mass of crude scraped pigment. The formula is: Extraction rate (%) = (mass of purified eumelanin/mass of crude scraped pigment) × 100%. We determined the protein content in the purified preparation. We used the Dumas combustion method. This method followed AOAC 992.15. We applied a nitrogen-to-protein conversion factor of 6.25. We determined fat content according to ISO 1443:1973. This standard expresses total fat as a percentage by mass. We performed all measurements in triplicate.

### 2.3 Structural characterization of GS eumelanin

#### 2.3.1 ICP-MS, SEM-EDS analyses of GS eumelanin

The detection of mineral elements in eumelanin from GS was performed in accordance with GB 5009.268–2016 (National Food Safety Standard: Determination of Multiple Elements in Food), and the analysis was carried out using inductively coupled plasma mass spectrometry (ICP-MS). The microstructure of the GS eumelanin was examined using a scanning electron microscope (SEM) (FEI Quanta FEG 650, JSM-IT800, JEOL Ltd., Tokyo, Japan). For SEM imaging, the GS eumelanin was sputter-coated with a thin layer of gold to enhance conductivity. The SEM was operated at an accelerating voltage of 1,000–3,000 kV, and images were captured at magnifications of 50 × and 150 × to observe the morphology and surface features of the samples.

Elemental composition was determined using energy-dispersive X-ray spectroscopy (EDS) coupled with the SEM system. The EDS (Ultra MAX 65, Oxford Instruments, Oxford, UK) analysis was performed under the same conditions as the SEM imaging, and the data were processed to identify the elemental composition of the sample surfaces.

#### 2.3.2 FTIR analysis of GS eumelanin

We freeze-dried the GS eumelanin, thoroughly mixed it with dry KBr powder at a mass ratio of 1:100, ground the mixture uniformly in an agate mortar, and then pressed it into a transparent pellet under a pressure of 10 tons for 2 min. We immediately placed the pellet in the sample chamber, performed scans over the wavenumber range of 4,000–400 cm^−1^ at a resolution of 4 cm^−1^, and recorded Fourier transform infrared spectroscopy (FTIR) using an INVENIO spectrometer (Bruker, Germany).

#### 2.3.3 UV–vis analysis of GS eumelanin

Ultraviolet–visible (UV–Vis) absorption spectra of GS eumelanin were recorded on an Evolution 201 spectrophotometer (Thermo Fisher Scientific, USA). The purified GS eumelanin was dissolved in 1%NaoH solution. The spectrum was recorded in the wavelength range of 200–800 nm using a standard quartz cuvette with a 1 cm path length. We used 1% NaOH solution as the blank reference.

#### 2.3.4 XPS analysis of GS eumelanin

X-ray photoelectron spectroscopy (XPS) analysis was performed using an ESCALAB 250Xi spectrometer (Thermo Fisher Scientific, USA) with monochromated Al Kα radiation (hν = 1486.6 eV). The X-ray source was operated at 150 W, with survey spectra recorded at a pass energy of 200 eV and high-resolution spectra at 30 eV. The base pressure in the analysis chamber was maintained below 1.0 × 10^−9^ mbar. Survey spectra were acquired over a binding energy range of 0–1,200 eV to determine the surface elemental composition. High-resolution spectra of C1s, N1s, and O1s were collected to identify the chemical states of constituent elements. All binding energies were calibrated using the adventitious carbon C1s peak at 284.8 eV as a reference. Peak fitting and data processing were performed using Thermo Scientific Avantage software.

### 2.4 Biological safety

#### 2.4.1 Cell viability

The inhibition rate of cell proliferation was evaluated using the CCK-8 assay. IEC-6 cells in the logarithmic growth phase were seeded into 96-well plates at 5 × 10^3^ cells per well and cultured in complete medium for 24 h to allow full adherence. The original medium was then removed and replaced with fresh medium containing GS eumelanin at concentrations of 1,000, 500, 200, 50, 10, and 1 μg/mL, or a control, and the cells were co-cultured for 24 to 48 h. At 24 and 48 h, 10 μL of CCK-8 solution was added to each well, and the plates were incubated in a cell culture incubator for 1 to 3 h in the dark. Finally, the absorbance of each well was measured at 450 nm using a microplate reader (Multiskan SkyHigh, Thermo, USA).

#### 2.4.2 Live-dead cell staining

Live-Dead Cell Staining was characterized using calcein-AM and propidium iodide double fluorescence staining. IEC-6 cells were seeded into 24-well plates and cultured in complete medium for 24 h to allow full adherence. The original medium was then removed and replaced with fresh medium containing GS eumelanin at concentrations of 1,000, 500, 200 μg/mL or a control, and the cells were co-cultured for 24 h. After co-culture, the cells were gently washed with pre-warmed PBS. A staining solution containing 2 μM calcein-AM and 4 μM propidium iodide was added, and the cells were incubated at 37 °C in the dark for 20 min. Following incubation, the cells were washed with PBS to remove excess dye and immediately observed under a confocal microscope. The green fluorescence of live cells was captured through the FITC channel, and the red fluorescence of dead cells was captured through the TRITC channel.

### 2.5 Experimental design and grouping

#### 2.5.1 Animal grouping

One hundred and fourteen male Wistar rats (approximately 250 g body weight) were purchased from Beijing Witte River Laboratory Animal Science and Technology Co., Ltd. (Beijing, China). The Wistar rat was selected as the experimental model due to its well-characterized physiological stability and metabolic similarity to humans. They were housed in controlled temperature (23 ± 1 °C), relative humidity (40–70%), and a 12-h light/dark cycle. The open-formula purified diets contained approximately 18% protein, 55% carbohydrate, and 4% fat by weight. They were fasted for 12 h before drug administration and watered ad libitum. To minimize potential confounders, the order of treatments and measurements was randomized. Additionally, the location of cages within the animal facility was rotated regularly to control for environmental gradients. All animal experiments have complied with the ARRIVE guidelines. Approval for the experimental protocol was obtained from the Animal Ethics Committee of Shaanxi University of Technology before the commencement of the study (Approval number 2025012301).

**Figure 1:**
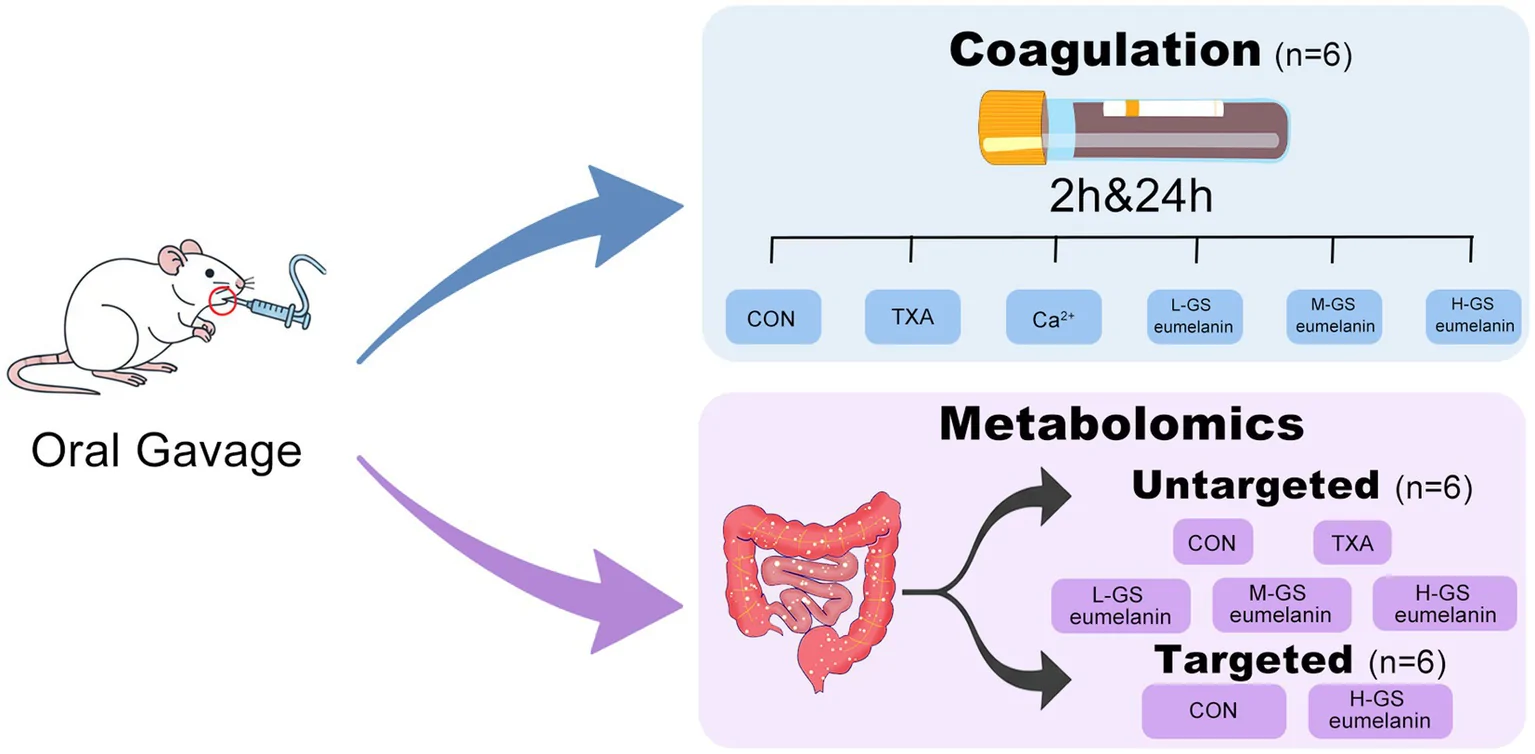
Schematic diagram of experimental rat grouping.

The study consisted of two sub-experiments (Figure 1). Sub-experiment 1 assessed coagulation function. It used 72 rats divided into 6 groups (*n* = 6). We tested at two time points: 2- and 24-h post-gavage. The groups were CON (saline, serving as the vehicle control), TXA (100 mg/kg), Ca^2+^ (60 mg/kg), L-GS eumelanin (400 mg/kg), M-GS eumelanin (800 mg/kg), and H-GS eumelanin (1,200 mg/kg). Sub-experiment 2 focused on metabolomics. It used 42 rats. For untargeted analysis, we used 30 rats divided into 5 groups (*n* = 6): CON, TXA, L-GS eumelanin, M-GS eumelanin, and H-GS eumelanin. For targeted PRM validation, we used the remaining 12 rats divided into 2 groups (*n* = 6): CON and H-GS eumelanin. All treatments were given via gavage in 5 mL saline.

#### 2.5.2 Dose selection and bias control

*Dose selection*. The doses of GS eumelanin (400, 800, and 1,200 mg/kg) were selected based on three considerations. First, the dose range was initially informed by the clinical dosage regimen of Haimo hemostatic capsules (The main component is eumelanin derived from the cuttlefish ink), with dose conversion from human to rat performed according to body surface area normalization. Second, the selected doses represent a low-medium-high gradient to allow for dose–response assessment. Third, the *in vitro* cytotoxicity assay (CCK-8) confirmed that high concentrations of GS eumelanin were non-cytotoxic to IEC-6 cells, providing an additional safety margin for the dose selection. These combined considerations ensured that the chosen doses were pharmacologically relevant, therapeutically informative, and toxicologically safe for the subsequent *in vivo* experiments.

*Sample size justification*. The sample size (*n* = 6/group) was determined based on power analysis assuming a large effect size (Cohen’s d = 1.2, *α* = 0.05, power = 0.80), which is consistent with the magnitude of differences observed in pilot studies. This yielded a minimum of 5–6 animals per group, which also aligns with standard practices in rodent coagulation and metabolomic studies. The study adhered to the 3R principles: Replacement - an *in vivo* model was necessary due to the systemic complexity of coagulation; Reduction - sample sizes were minimized to the statistical requirement with no extraneous animals; Refinement - standardized gavage, environmental enrichment, and blinded assessments were implemented to minimize stress and variability.

*Randomization and blinding were performed*. Before treatment, all rats were assigned to experimental groups. The assignment used a computer-generated random number sequence from the RAND function in Excel. For coagulation assessment, one investigator did the gavage. Another investigator did the tail bleeding time and coagulation index measurements. The latter remained blinded to group allocation throughout. For metabolomics, technicians prepared samples and acquired LC–MS/MS data. They were also blind to group identities. Group codes were revealed only after all data collection and primary statistical analyses were finished.

### 2.6 Identification of key GS eumelanin intermediate metabolites by UHPLC-Q-Orbitrap HRMS analysis

#### 2.6.1 Sample pretreatment for the detection of GS eumelanin metabolites

The tails of forty-two rats were amputated 3 cm from the tip. Two hours post-treatment, all animals were deeply anesthetized via intraperitoneal injection of tribromoethanol (2.5%, 8 mL/kg). After confirming the loss of pedal withdrawal reflex, euthanasia was performed by exsanguination via cardiac puncture. The absence of a heartbeat, respiratory movements, and bilateral pupil dilation further confirmed death. Subsequently, a 5 cm segment of the small intestine was collected for further analysis. Take out 0.5 mL of the small-intestinal contents, soak them in 10 mL of 30% acetonitrile (containing 1% formic acid), and subject them to ultrasonication (KQ-250 DA, Kunshan Ultrasonic Instrument Co., Ltd., Jiangsu, China) at 4 °C for 30 min. To ensure complete recovery of GS eumelanin metabolites, this extraction was repeated twice. The supernatants from both extractions were combined and centrifuged at 4 °C for 15 min at 8,944 g before further analysis.

#### 2.6.2 Untargeted metabolomics analysis of GS eumelanin metabolites in gastrointestinal tracts

All samples were analyzed using ultrahigh-performance liquid chromatography quadrupole-Orbitrap high-resolution mass spectrometry (UHPLC-Q-Orbitrap HRMS) (Thermo Scientific, Waltham, MA, USA), equipped with a heated electrospray ionization (HESI) source. Chromatographic separations were performed on an ACQUITY UPLC® HSS T3 column (2.1 mm × 100 mm, 1.8 μm, Waters, MA, USA), with column temperature maintained at 30 °C and the flow rate of acetonitrile (phase A) and 0.1% formic acid in water (phase B) at 0.2 mL/min. The gradient elution scheme was as follows: from 0 to 10 min, 2% A; from 10 to 20 min, 2–20% A; from 20 to 25 min, 20–50% A; from 25 to 27 min, 50–90% A; from 27 to 30 min, 90–2% A. The injection volume was 5 μL.

Positive and negative ion mode HESI sources were used. The electrospray source parameters were set as follows: a spray voltage of 3.5 kV in positive modes and 2.5 kV in negative modes; scanning range, *m/z* 70–1,000; collision energy, 20 eV; ion pipe temperature, 320 °C; sheath gas flow rate, 30 psi; and auxiliary gas flow rate, 10 psi.

Untargeted metabolomic profiling of small intestinal GS eumelanin metabolites was conducted using UHPLC-Q-Orbitrap HRMS in both positive and negative ion modes, utilizing a dual scanning strategy with Full MS and Full MS/dd-MS^2^. The raw MS data from the control group, experimental groups, and TXA-treated groups were processed using Compound Discoverer 3.2 software (Thermo Fisher Scientific, USA), which incorporated background subtraction protocols. Compound identification was achieved using multi-database matching (mzCloud and ChemSpider) with strict confidence criteria. The metabolomic data were subjected to log2 transformation and Pareto scaling to improve normality and variance homogeneity, followed by unsupervised principal component analysis (PCA). ANOVA was employed to ascertain statistical significance, and the results were subsequently corroborated using the targeted quantification of specific metabolites.

#### 2.6.3 Targeted metabolomics analysis of GS eumelanin metabolites in the gastrointestinal tracts

In the targeted metabolomics analysis of small intestinal metabolites of GS eumelanin, we used standard substances of 20 amino acids, TXA, and ACA. To eliminate potential interferences from the other compounds in the sample, compounds present in the solvent used for sample preparation were subtracted. This procedure was applied to both CON and H-GS eumelanin. Full MS and PRM scanning modes were utilized to conduct a precision analysis of the 20 amino acids, TXA, and ACA in GS eumelanin metabolites. Chromatographic separations were performed on a HILIC column (150 mm × 2.1 mm, 2.6 μm, Thermo Scientific, USA), with column temperature maintained at 30 °C, and the flow rate of acetonitrile (phase A) and 0.1% formic acid water (phase B) was set at 0.2 mL/min. The gradient elution scheme was as follows: 0–5 min, 100–70% A; 5–15 min, 70% A; 15–20 min, 70–50% A; 20–23 min, 50–100% A; 23–25 min, 100% A. The injection volume was 5 μL.

Quality control in metabolomics analysis. To ensure data reliability, the following procedures were implemented: (1) QC samples (pooled from equal volumes of all samples) were injected every 10 runs to monitor instrument performance and batch-to-batch variability; (2) injection order was randomized across batches to control batch effects; (3) metabolites were identified by matching retention times, exact mass *m/z* (to five decimal places), fragment ion *m/z*, and specific PRM transitions against authentic standards, corresponding to Metabolomics Standards Initiative level 1; and (4) blank samples were processed in parallel to exclude contamination from reagents or extraction procedures.

We performed a recovery experiment using the standard addition method. We took the GS eumelanin intestinal metabolic samples. These samples had known analyte contents. We then added a mixed standard solution of TXA and ACA. The preparation followed the procedure described in Section 2.6.1. All samples were processed in triplicate. We calculated the recovery rate and the relative standard deviation (RSD) using the formula below:

Recovery (%) = (Measured amount after spiking − Original sample content) / Amount of standard added × 100%.

The optimal mass spectrometry parameters for TXA, ACA, and several key amino acids, including molecular ions, important fragment ions, and ion monitoring modes, are listed in Supplementary Table S1.

### 2.7 Hemorrhage and coagulation parameter measurements

All procedures were performed under deep anesthesia induced by intraperitoneal injection of tribromoethanol (2.5%, 8 mL/kg). After confirming the loss of the pedal withdrawal reflex, the rat’s tail was severed 3 cm from the tip. Blood outflow was monitored by aspirating blood with pre-weighed gauze every 5 s until a clot formed, and the hemostasis time was recorded. Hemorrhage volume was quantified by weighing the gauze before and after blood aspiration.

Immediately following the tail amputation procedure, a terminal blood collection of 1.8 mL was performed via cardiac puncture and mixed with 0.2 mL of sodium citrate (183.5 mM) as an anticoagulant. The blood sample was centrifuged at 3,000 × g for 15 min at 25 °C to obtain plasma. Plasma prothrombin time (PT), activated partial thromboplastin time (APTT), thrombin time (TT), and fibrinogen (FIB) levels were quantified using an automated coagulation analyzer within 3 h of collection. After blood collection, animals were euthanized by exsanguination, followed by confirmation of death through the absence of heartbeat and respiratory movements.

Humane endpoints were predefined as follows: >20% body weight loss, inability to ambulate, self-mutilation of the wound site, or failure to eat/drink for >24 h. Animals meeting these criteria would be humanely euthanized via CO₂ inhalation followed by cervical dislocation. No animal reached these endpoints, so no early termination was needed.

### 2.8 Statistical analysis

All experimental data were presented as mean ± SD (n = 6). Statistical analyses were performed using one-way analysis of variance (ANOVA) followed by Duncan’s multiple range test (SPSS 20.0). We set significance at *p* < 0.05, high significance at *p* < 0.01, and extreme significance at *p* < 0.001. Hierarchical clustering analysis heatmaps and bar charts for coagulation index analysis were generated using Origin 2022 software. Enrichment and pathway analyses were performed and summarized with MetaboAnalyst 5.0.

## 3 Results

### 3.1 GS eumelanin is an amorphous solid

On a dry weight basis, the extraction rate of GS eumelanin from the crude scraped pigment was 6.24% (w/w). The product had a purity of 97.7% (w/w), with 1.45% protein and 0.83% fat. These results confirm the effectiveness of the purification procedure. This procedure removed most of the protein and lipid contaminants from the crude pigment extract. As a result, we obtained a high-purity product. This product is suitable for subsequent biological evaluation.

**Figure 2:**
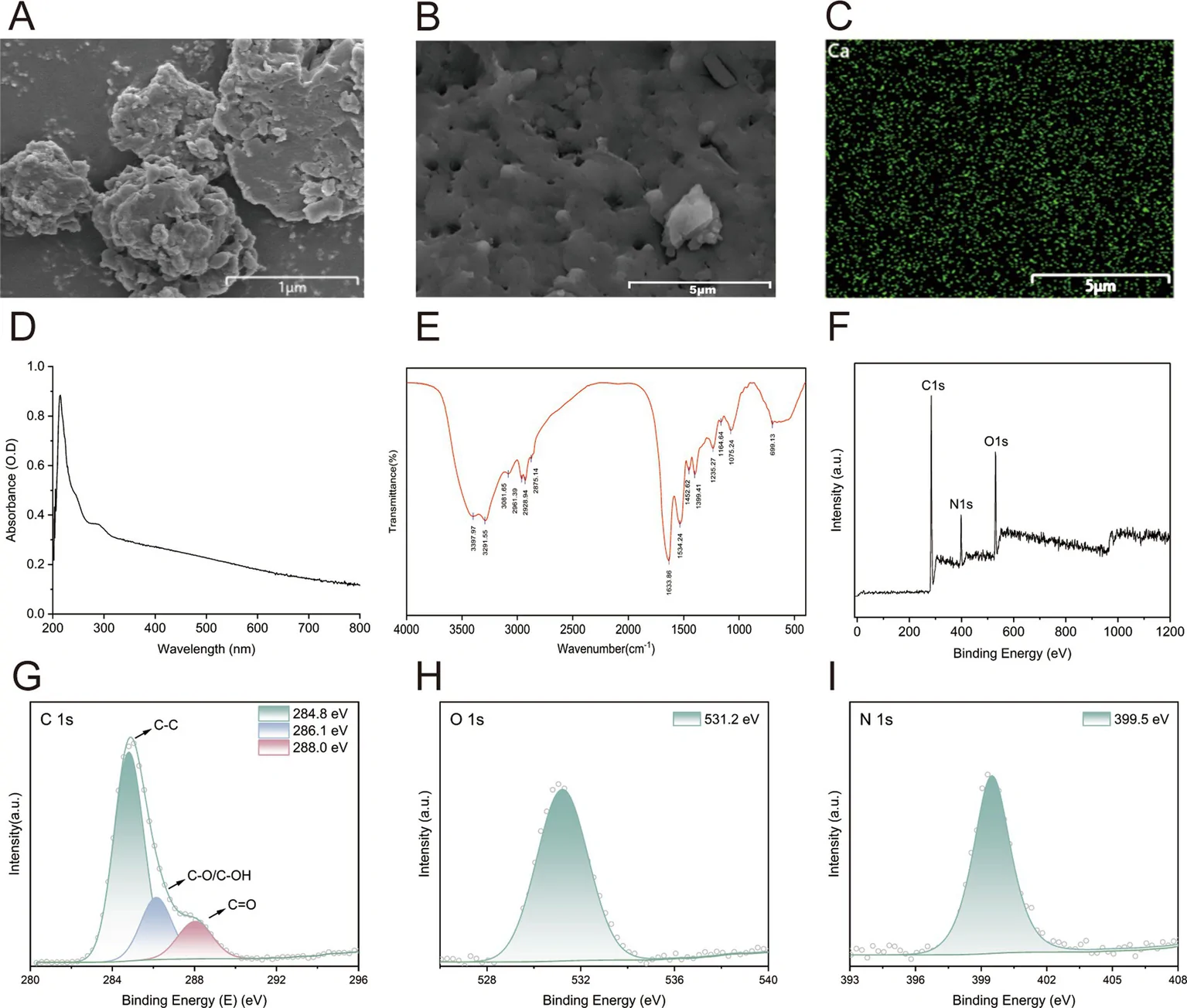
Structural characterization of GS eumelanin. **(A)** The SEM micrographs of GS eumelanin, with scale bars representing 1 μm. **(B)** The SEM micrographs of GS eumelanin, with scale bars representing 5 μm. **(C)** In the SEM-EDS elemental mapping, with scale bars representing 5 μm, the green represents the EDS Ca^2+^ map of GS eumelanin. **(D)** UV–visible absorbance spectrum of GS eumelanin. **(E)** FTIR spectrum of GS eumelanin. **(F)** XPS survey spectrum showing C1s, O1s, and N1s peaks with no detectable heavy metals. **(G)** C1s spectrum deconvoluted into C-C (284.8 eV), C-O/C-OH (286.1 eV), and C=O (288.0 eV). **(H)** O1s spectrum at ~531.2 eV (C=O). **(I)** N1s spectrum at ~399.5 eV (pyrrole-type N/amide groups).

The microstructure of GS eumelanin was examined using SEM-EDS analysis (Figures 2A–C). The results indicate that GS eumelanin did not exhibit well-defined structural units; instead, it appeared as irregular agglomerates. SEM-EDS analysis further revealed that GS eumelanin is a type of bio-metal–organic framework (Bio-MOF). The metal ions present were primarily Ca^2+^, and ICP-MS analysis determined that the Ca^2+^ content was 60.06 ± 0.76 mg/g.

The UV–Vis absorption spectrum of GS eumelanin is shown in Figure 2D. The absorption intensity gradually decreased with increasing wavelength from the UV region to the visible region (400–800 nm), without any distinct absorption peaks in the visible range.

The alkaline hydrolysate of GS eumelanin was analyzed using FTIR spectroscopy (Figure 2E). The obtained spectrum exhibits a broad and intense absorption peak near 3,397 cm^−1^, which was attributed to the overlapping of O-H and N-H stretching vibrations. This feature indicates the presence of hydroxyl, phenolic hydroxyl, and indole imino groups, along with an inter- or intramolecular hydrogen-bonding network-a characteristic signature of the polyphenolic/indolic polymer structure of eumelanin. A strong absorption band at 1633 cm^−1^ was observed, possibly arising from amide I C=O stretching, asymmetric stretching of carboxylate (COO^−^), or C=C skeletal vibrations of indole rings, suggesting the existence of carbonyl and aromatic moieties. The absorption near 1,534 cm^−1^ was primarily assigned to C=N and C=C skeletal stretching of indole rings, with possible contributions from N-H in-plane bending, further supporting the presence of indole or related nitrogen-containing structures. While bands at 1452 and 1,399 cm^−1^ corresponded to CH₂/CH₃ bending vibrations, likely originating from alkyl chains in melanin biosynthetic precursors. The band near 1,235 cm^−1^ was assigned to C-N and C-O stretching, consistent with indole and phenolic hydroxyl structures. Absorption features in the 1,164 and 1,075 cm^−1^ range were mainly due to C-O stretching, providing additional evidence for phenolic hydroxyl, ether, or carboxylate groups. Furthermore, the absorption at 699 cm^−1^, characteristic of out-of-plane C-H bending of aromatic rings, further confirmed the presence of an aromatic heterocyclic system.

Characteristic vibrations corresponding to O-H/N-H, C=O/C=C, C-N, and C-O groups were observed in the overall spectrum. These spectral features are consistent with the typical polyindole structure and the oxygen- and nitrogen-containing functional group composition of eumelanin. The findings indicate that GS eumelanin exhibited pronounced indole polymer characteristics.

The surface elemental composition and chemical states of GS eumelanin were analyzed using XPS. The survey spectrum revealed three major peaks corresponding to C1s (~284.8 eV), O1s (~531.2 eV), and N1s (~399.5 eV), with relative atomic percentages of 72.77, 16.19, and 11.05%, respectively (Figure 2F). The high-resolution C1s spectrum was deconvoluted into three peaks at 284.8 eV, 286.1 eV, and 288.0 eV, assigned to C-C, C-O, and C=O, respectively (Figure 2G). The O1s spectrum showed peaks at approximately 531.2 eV, corresponding to C=O (carbonyl/carboxyl) species (Figure 2H). The N1s spectrum exhibited a main peak at approximately 399.8 eV (Figure 2I).

### 3.2 Biosafety analysis

**Figure 3:**
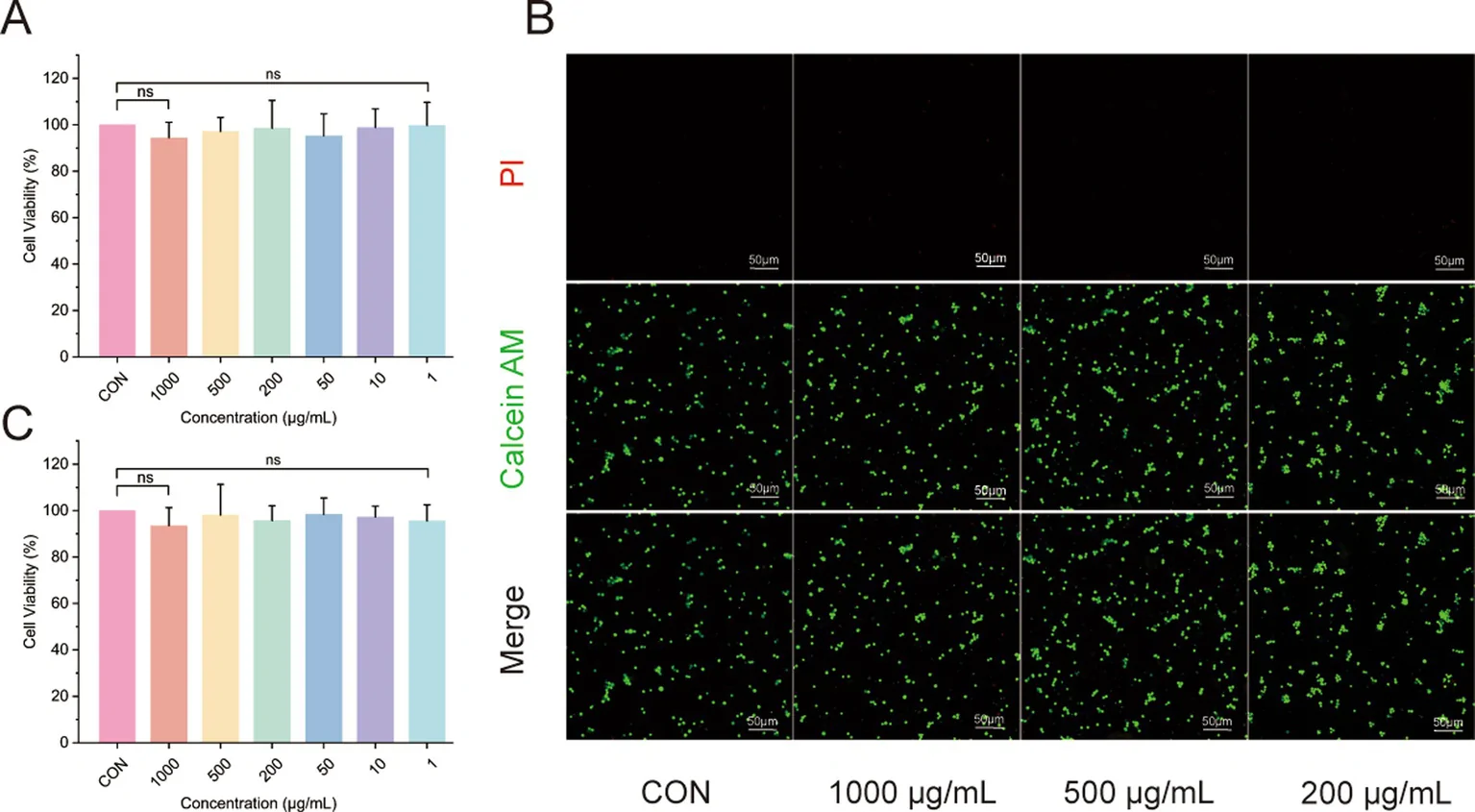
Biosafety analysis of GS eumelanin. **(A)** The cytotoxic effect of GS eumelanin on IEC-6 after 24 h of treatment was determined using the CCK-8 assay. **(B)** Live/dead fluorescence staining of IEC-6 after GS eumelanin intervention. **(C)** The cytotoxic effect of GS eumelanin on IEC-6 after 48 h of treatment was determined using the CCK-8 assay. Statistical significance is as follows: ns: *p* > 0.05, *n* = 6.

IEC-6 cells were treated with GS eumelanin at different concentrations (1,000, 500, 200, 50, 10, and 1 μg/mL), and the results show that GS eumelanin did not affect cell viability at 24 and 48 h after application (Figures 3A,C).

Fluorescence microscopy reveals that cells cultured on GS eumelanin exhibited comparable green fluorescence intensity and density to the control group, with nearly all cells displaying viable morphology and intact nuclei. PI-positive dead cells were rarely observed (<5%) in both groups (Figure 3B). This observation indicates that GS eumelanin has good biological safety. For *in vivo* safety, all animals were closely monitored during the acute experimental period (2 h and 24 h post-gavage) for any signs of distress or mortality; no adverse events were observed before terminal blood collection.

### 3.3 UHPLC-Q-Orbitrap HRMS analysis

#### 3.3.1 Untargeted metabolomic analysis

H-GS eumelanin was dissolved in 10 mL of normal saline and administered via intubation into the stomachs of the rats. Two hours later, the rats were euthanized. Black Bio-MOFs nanoparticles were found in the stomach and a small amount in the duodenum. No black Bio-MOFs nanoparticles were detected in the jejunum or ileum. Therefore, we analyzed the metabolic products from the middle parts of the small intestine (jejunum and ileum) 2 h after H-GS eumelanin was intubated into the stomachs of the rats.

**Figure 4:**
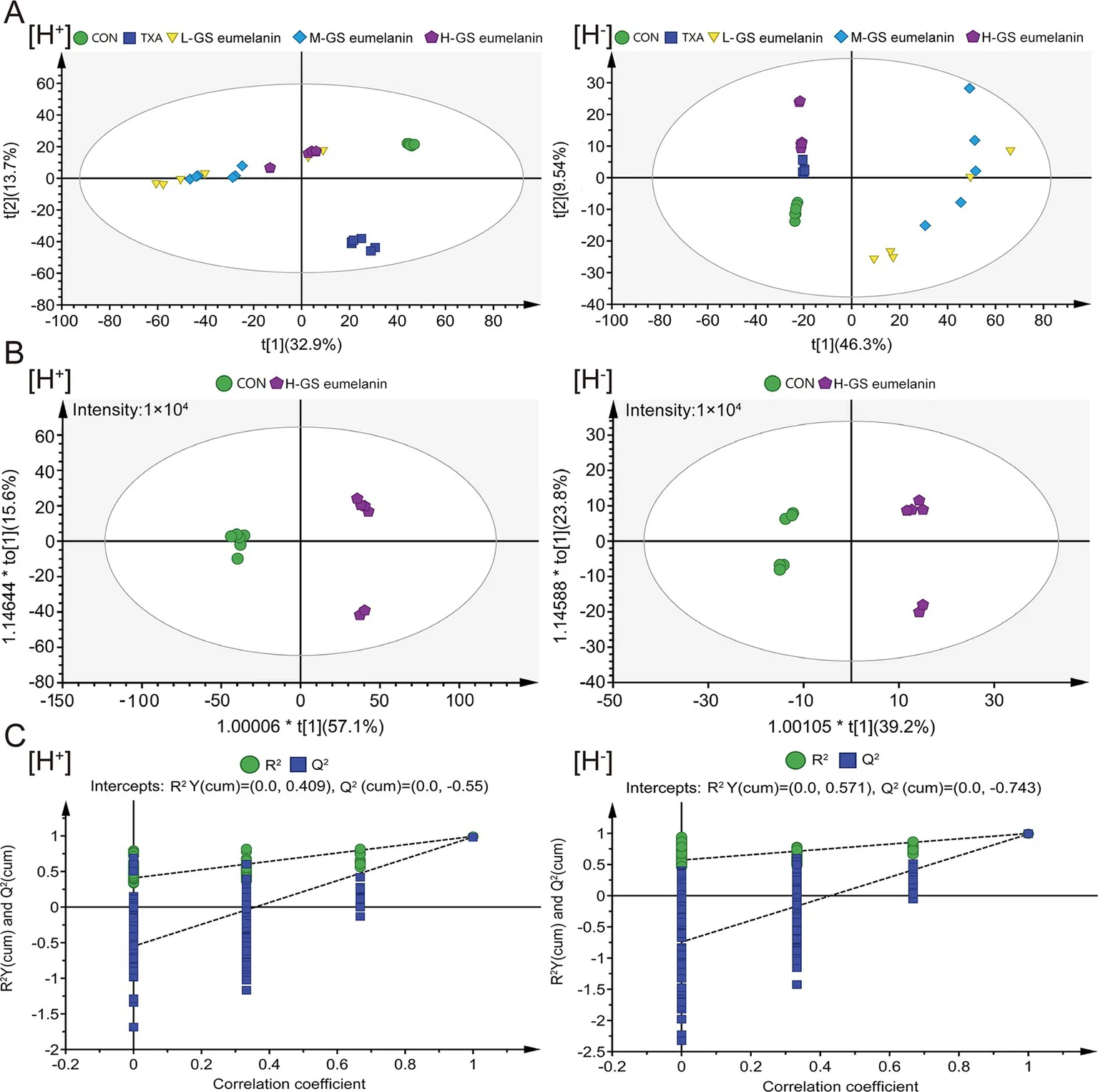
Untargeted metabolomics analysis results of CON, TXA, L-GS eumelanin, M-GS eumelanin, and H-GS eumelanin. **(A)** Score scatter plot of the principal component analysis (PCA) using positive and negative ion scanning mode models. **(B)** Score scatter plot of orthogonal partial least squares-discriminant analysis (OPLS-DA) for CON vs. H-GS eumelanin using positive and negative ion scanning modes. **(C)** Permutation plot test of orthogonal partial least squares-discriminant analysis (OPLS-DA) for CON vs. H-GS eumelanin using positive and negative ion scanning modes. CON: Control group; TXA: Positive administration group; L-GS eumelanin: Low-dose group; M-GS eumelanin: Medium-dose group; H-GS eumelanin: High-dose group.

Untargeted metabolomics analysis of the CON, TXA, L-GS eumelanin, M-GS eumelanin, and H-GS eumelanin groups was conducted using UHPLC-Q-Orbitrap HRMS in Full MS and Full MS/dd-MS^2^ scanning modes, with both positive and negative ion modes. The Principal Component Analysis (PCA) results for both ion modes are shown in Figure 4A, demonstrating a high degree of similarity and a clustering trend among the groups.

Principal component 1 (t1) of intestinal metabolites shows a significant difference between the CON group and all three GS eumelanin dose groups in positive ion mode. The H-GS eumelanin group was closer to the TXA group compared to the other dose groups. Principal component 2 (t2) revealed significant differences between the CON and TXA groups in both positive and negative-ion modes. Therefore, we focused on differences in small intestinal metabolites between the CON and H-GS eumelanin groups. As shown in Figure 4B, in positive ion mode, the x-axis accounted for 57.1% and the y-axis for 15.6%. In negative ion mode, the x-axis accounted for 39.2% and the y-axis for 23.8%. Both modes show clustering within the CON and H-GS eumelanin groups. However, a clear distinction was observed between the CON and H-GS eumelanin groups.

Orthogonal partial least squares discriminant analysis (OPLS-DA) permutation tests show R^2^Y (cum) values of 0.571 ([H]^+^) and 0.409 ([H]^-^), and Q^2^ (cum) values of 0.743 and 0.55 (Figure 4C). We found that both goodness-of-fit parameters (R^2^ and Q^2^) calculated for the ranked data were lower than the corresponding original points on the right-hand side (1 on the X-axis). Additionally, the intercepts of the Q^2^ regression lines were all below zero, indicating minimal overfitting in the original prediction model. These results suggest that the separation model is statistically valid, and we used these data for further analysis.

#### 3.3.2 Targeted metabolomic analysis

**Figure 5:**
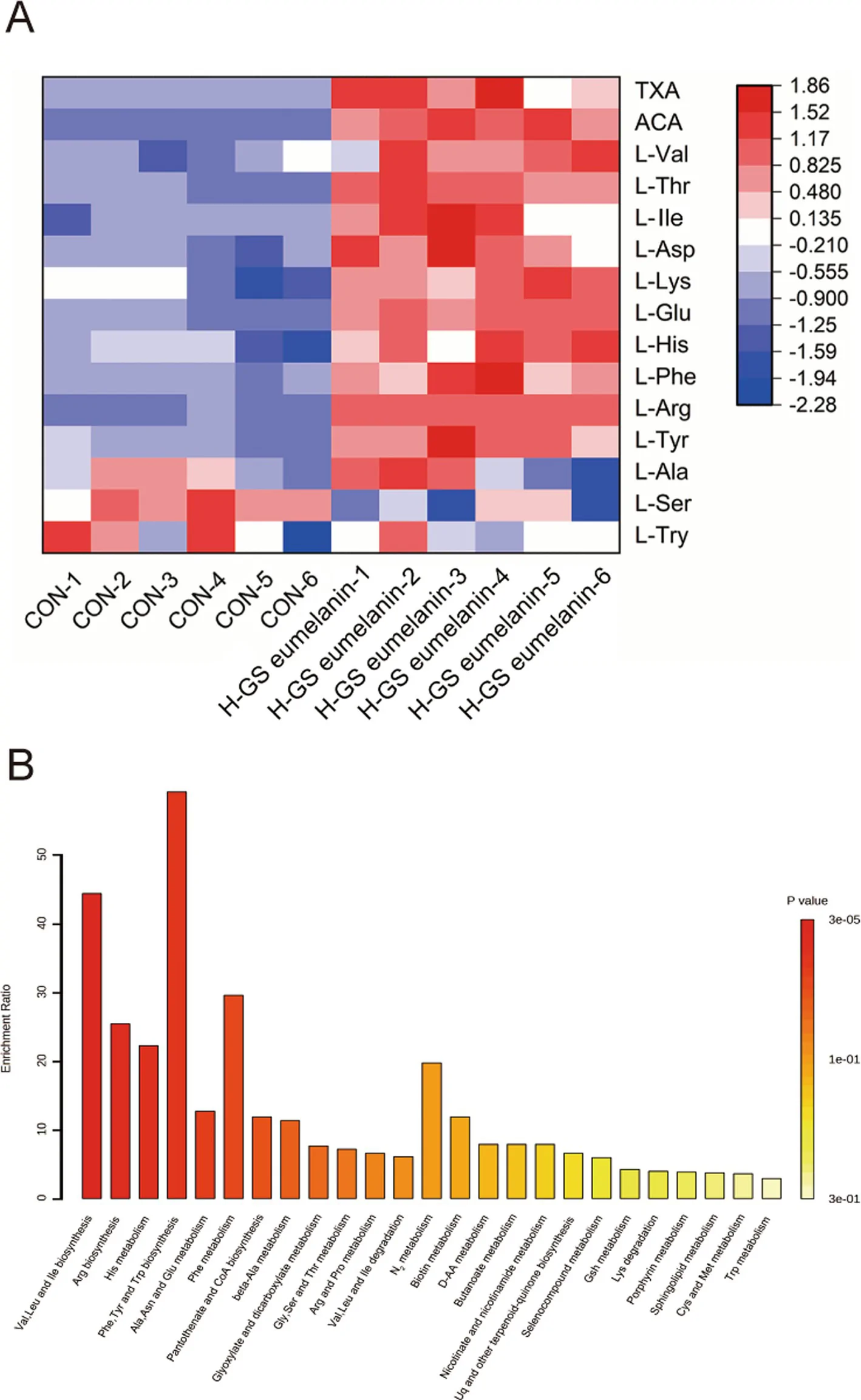
Targeted metabolomics analysis results of CON and H-GS eumelanin. **(A)** Heatmap of hierarchical clustering analysis of differences between the CON and H-GS eumelanin. **(B)** The enrichment analysis and pathway analysis summary of CON and H-GS eumelanin were conducted using MetaboAnalyst 5.0. CON: Control group; H-GS eumelanin: High-dose group; TXA: Positive administration group; ACA: 6-Aminocaproic acid; Val: Valine; Thr: Threonine; Ile: Isoleucine; Asp: Aspartic acid; Lys: Lysine; Glu: Glutamate; His: Histidine; Phe: Phenylalanine; Arg: Arginine; Tyr: Tyrosine; Ala: Alanine; Ser: Serine; Try: Tryptophan.

Based on untargeted metabolomics analysis, we identified that the main differences in intestinal metabolites between the CON and H-GS eumelanin groups were nitrogen-containing organic compounds. To ensure data accuracy, we conducted targeted metabolomics analysis of these compounds using PRM scanning mode. The compounds were identified by their molecular ion and three fragment ion peaks in both positive- and negative-ion modes, and subsequently validated using authentic standards (MSI level 1). Heatmaps were generated from compound peak areas to show differences between the CON and H-GS eumelanin groups. As shown in Figure 5A, the heatmaps display the results of the clustering analysis on the differential metabolites. GS eumelanin was converted into amino acids and their structural analogs in the small intestine. Compared with the CON group, TXA and ACA were detected in the small intestine following GS eumelanin administration, accompanied by significant upregulation of valine, threonine, isoleucine, aspartic acid, lysine, glutamate, histidine, phenylalanine, arginine, and tyrosine, and significant downregulation of serine and tryptophan (Supplementary Table S2).

The extraction recoveries of TXA and ACA from the intestinal matrix were determined to be 95.95 ± 3.27% and 94.64 ± 2.97% (mean ± SD, n = 3), respectively, with relative standard deviations (RSD) below 10%, indicating that the sample preparation method was reliable and reproducible for both targeted analytes (Supplementary Table S3).

Finally, significant metabolites, along with amino acids and their structural analogs, were mapped to biochemical pathways using metabolic pathway and enrichment analysis. KEGG pathway analysis was performed on the MetaboAnalyst platform, identifying 25 metabolic pathways in the small intestines of the CON and H-GS eumelanin-treated groups (Figure 5B). The seven pathways with the highest enrichment of differential metabolites were: (1) valine, leucine, and isoleucine biosynthesis; (2) arginine biosynthesis; (3) histidine metabolism; (4) phenylalanine, tyrosine, and tryptophan biosynthesis; (5) alanine, asparagine, and glutamate metabolism; (6) phenylalanine metabolism; (7) pantothenate and coenzyme A biosynthesis.

### 3.4 Hemorrhage volume and hemostasis time analysis

**Figure 6:**
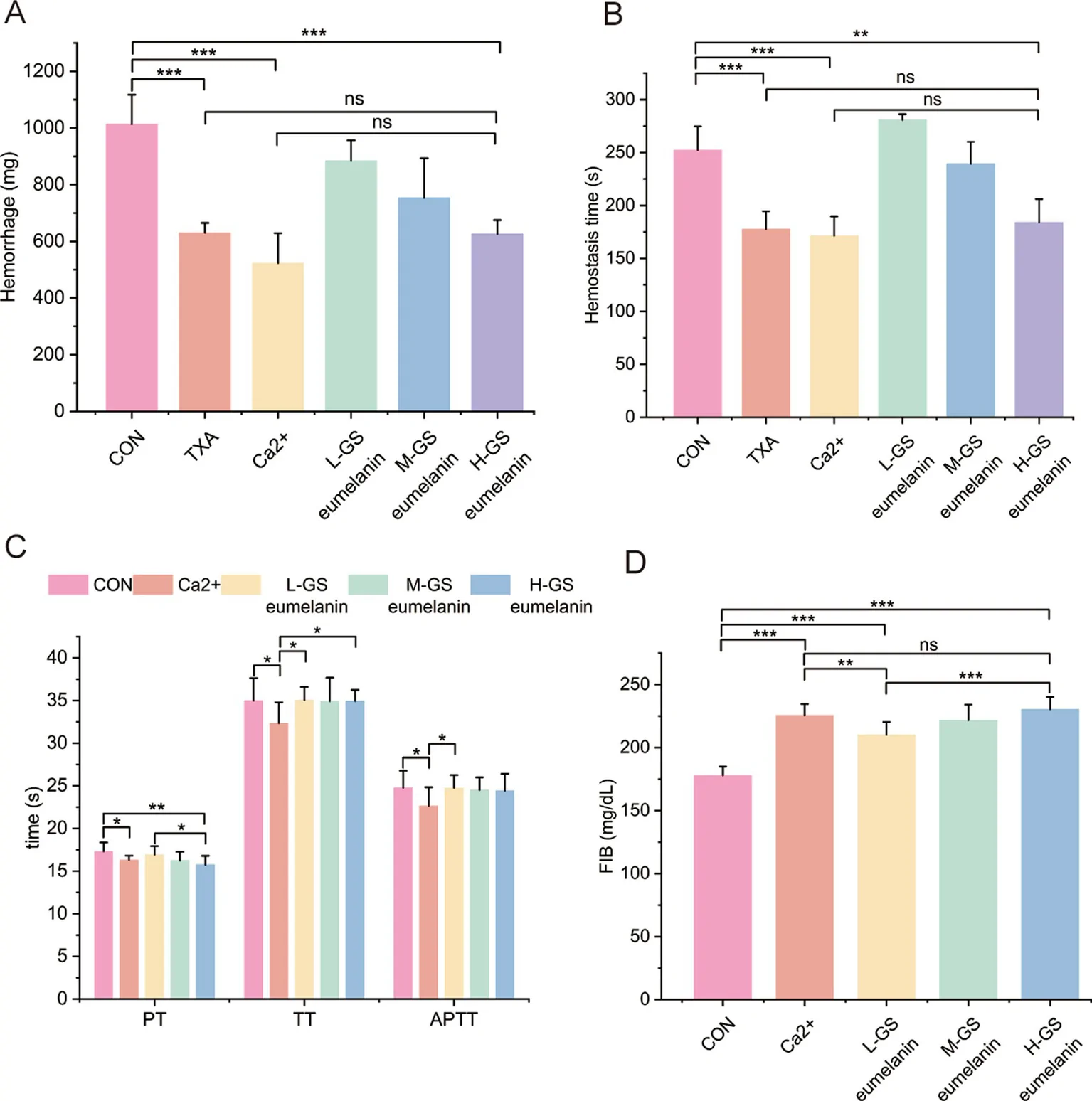
Coagulation profile 2 h after gavage of GS eumelanin administration in rats. *In vivo* hemostasis analysis results for CON, TXA, Ca^2+^, L-GS eumelanin, M-GS eumelanin, and H-GS eumelanin. **(A)** Hemorrhage volume measured in CON, TXA, Ca^2+^, L-GS eumelanin, M-GS eumelanin, and H-GS eumelanin. **(B)** Hemostasis time recorded for CON, TXA, Ca^2+^, L-GS eumelanin, M-GS eumelanin, and H-GS eumelanin. **(C)** PT, TT, and APTT analysis results for CON, Ca^2+^, L-GS eumelanin, M-GS eumelanin, and H-GS eumelanin. **(D)** FIB levels in CON, Ca^2+^, L-GS eumelanin, M-GS eumelanin, and H-GS eumelanin. Statistical significance is as follows: Ns: *p* > 0.05, *p < 0.05, **p < 0.01, ***p < 0.001, n = 6.

**Figure 7:**
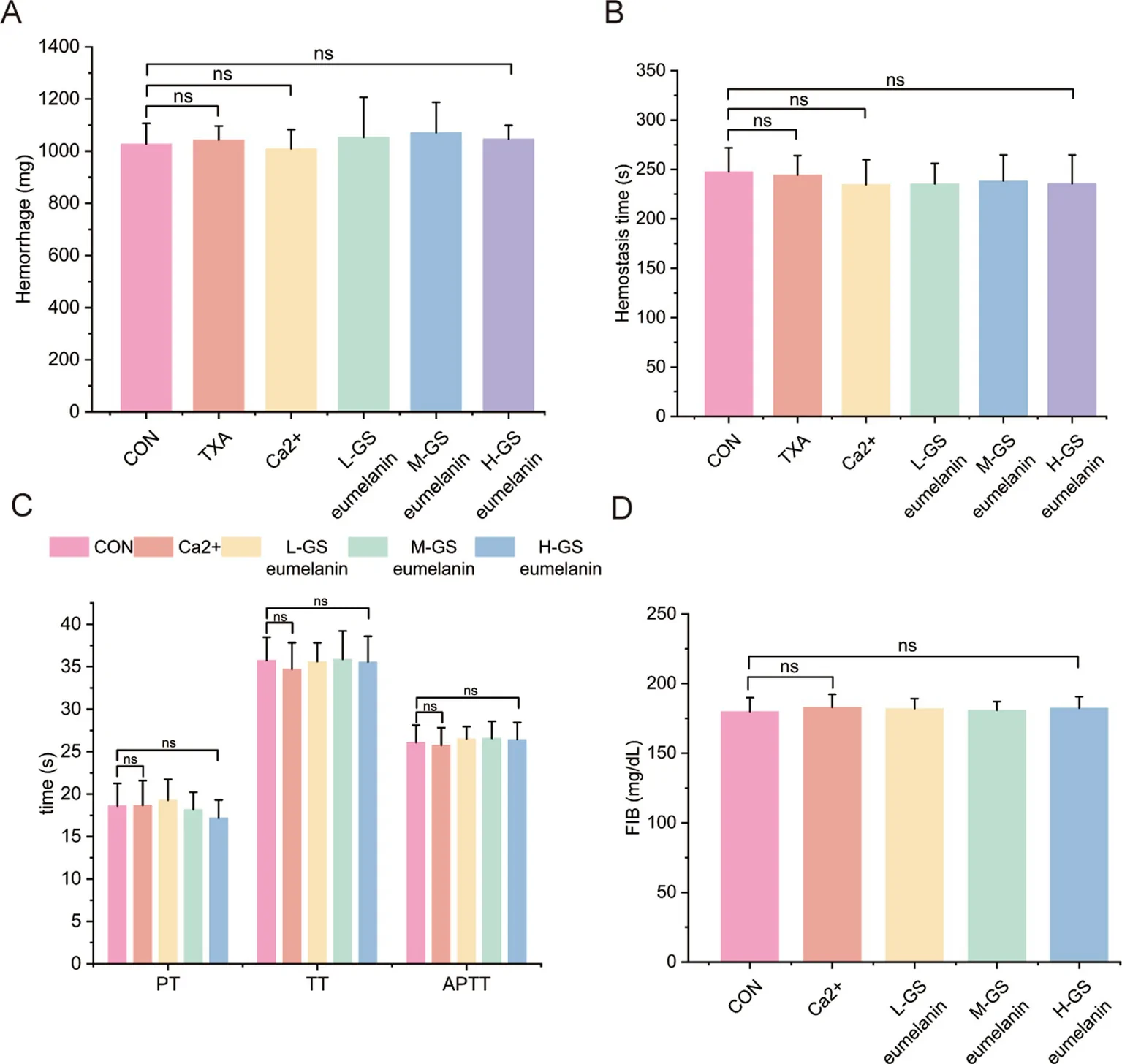
Coagulation profile 24 h after gavage of GS eumelanin administration in rats. In vivo hemostasis analysis results for CON, TXA, Ca^2+^, L-GS eumelanin, M-GS eumelanin, and H-GS eumelanin **(A)** Hemorrhage volume measured in CON, TXA, Ca^2+^, L-GS eumelanin, M-GS eumelanin, and H-GS eumelanin **(B)** Hemostasis time recorded for CON, TXA, Ca^2+^, L-GS eumelanin, M-GS eumelanin, and H-GS eumelanin **(C)** PT, TT, and APTT analysis results for CON, Ca^2+^, L-GS eumelanin, M-GS eumelanin, and H-GS eumelanin **(D)** FIB levels in CON, Ca^2+^, L-GS eumelanin, M-GS eumelanin, and H-GS eumelanin. Statistical significance is as follows: *s*: *p* > 0.05, *p < 0.05, **p < 0.01, ***p < 0.001, *n* = 6.

The hemorrhage volume in rats after gavage of TXA, Ca^2+^, and the three doses of GS eumelanin was lower than that observed in the CON after 2 h (Figure 6A). TXA (630 ± 35 mg), Ca^2+^ (523 ± 106 mg), and H-GS eumelanin (626 ± 49 mg) differ extremely significantly from the CON (1,013 ± 104 mg) (*p* < 0.001). However, the difference between TXA (630 ± 35 mg) and H-GS eumelanin (626 ± 49 mg), as well as between Ca^2+^ (523 ± 106 mg) and H-GS eumelanin (626 ± 49 mg), was not statistically significant. (*p* > 0.05). However, the hemorrhage volume in rats 24 h after administration showed no significant difference compared with the CON group (*p* > 0.05) (Figure 7A).

The hemostasis time in rats after gavage with TXA, Ca^2+^, M-GS eumelanin, and H-GS eumelanin was lower than in the CON group at 2 h (Figure 6B). TXA (178 ± 17 s) and Ca^2+^ (171 ± 18 s) show extremely significant differences compared to the CON (252 ± 22 s) (*p* < 0.001). H-GS eumelanin (184 ± 22 s) shows highly significant differences compared to CON (252 ± 22 s) (95% CI: −101 to −35 s, *p* < 0.01). There was no significant difference between TXA (178 ± 17 s) and H-GS eumelanin (184 ± 22 s) (*p* > 0.05), nor between Ca^2+^ (171 ± 18 s) and H-GS eumelanin (184 ± 22 s) (*p* > 0.05). However, the hemostasis time in rats 24 h after administration showed no significant difference compared with the CON group (*p* > 0.05) (Figure 7B).

At 2 h post-gavage, the H-GS eumelanin group showed a 38.2% reduction in hemorrhage volume and a 27.0% reduction in hemostasis time compared with the CON group (*p* < 0.01). As the GS eumelanin dose increased, both hemorrhage volume and hemostasis time decreased. These results show that all three doses of GS eumelanin effectively lowered blood loss in rats.

### 3.5 Analysis of four indicators of coagulation

PT, APTT, TT, and FIB are key parameters for clinical coagulation screening, as well as for laboratory studies and the safety evaluation of hemostatic or coagulation drugs. We evaluated the safety of GS eumelanin by measuring PT, APTT, TT, and FIB.

Two hours after administration, the PT values in the Ca^2+^ and three GS eumelanin dose groups were lower than those in the CON group. A statistically significant difference in PT was observed between the Ca^2+^ (16.3 ± 0.5 s) and CON (17.3 ± 1.1 s) (*p* < 0.05). The H-GS eumelanin (15.8 ± 1.0 s) showed a highly significant difference in PT value compared to the CON (17.3 ± 1.1 s) (95% CI: −2.5 to −0.6 s, Cohen’s d = 1.43, *p* < 0.01), as well as a statistically significant difference when compared with the L-GS eumelanin (16.9 ± 1.0 s) (*p* < 0.05). In terms of TT, a significant difference was found between the Ca^2+^ (32.3 ± 2.4 s) and CON (35.0 ± 2.7 s) (*p* < 0.05). Compared to the Ca^2+^ (32.3 ± 2.4 s), both L-GS eumelanin (35.1 ± 1.5 s) and H-GS eumelanin (34.9 ± 1.3 s) also exhibited significant differences in TT values (*p* < 0.05). In the APTT assay, significant differences were detected between the Ca^2+^ (22.6 ± 2.2 s) and CON (24.8 ± 2.0 s), and between the L-GS eumelanin (24.8 ± 1.5 s) and Ca^2+^ (22.6 ± 2.2 s) (*p* < 0.05) (Figure 6C). Regarding FIB levels, the values in the Ca^2+^ (225 ± 9 mg/dL), L-GS eumelanin (210 ± 10 mg/dL), and H-GS eumelanin (230 ± 10 mg/dL) differed extremely significantly from those in the CON (178 ± 7 mg/dL) group (95% CI: 42 to 62 mg/dL, Cohen’s d = 6.02, *p* < 0.001). Specifically, the FIB value of the Ca^2+^ (225 ± 9 mg/dL) showed a highly significant difference compared to that of the L-GS eumelanin (210 ± 10 mg/dL) (*p* < 0.01), but no significant difference was observed when compared with the H-GS eumelanin (230 ± 10 mg/dL) (*p* > 0.05). In contrast, an extremely significant difference was found between the L-GS eumelanin (210 ± 10 mg/dL) and H-GS eumelanin (230 ± 10 mg/dL) (*p* < 0.001) (Figure 6D).

Among the three GS eumelanin dose groups, dose-dependent trends were observed for PT (16.9 ± 1.0 s for L-GS eumelanin, 16.2 ± 1.0 s for M-GS eumelanin, and 15.8 ± 1.0 s for H-GS eumelanin) and FIB (210 ± 10, 221 ± 12, and 230 ± 10 mg/dL, respectively), suggesting a biological gradient of effect for these two parameters. Twenty-four hours after administration, there were no significant differences in APTT, TT, FIB, and PT among the groups (Figures 7C, D).

## 4 Discussion

### 4.1 GS eumelanin exhibits an irregular aggregation structure

Although eumelanin has been widely studied (22), the characterization of eumelanin derived from the skin of GS has not been reported. In this study, the GS eumelanin did not form well-defined structural units but instead showed irregular aggregated structures. This morphology differs from the nearly spherical nanoparticles usually produced by cuttlefish eumelanin (23). Eumelanin consists of basic structural units, such as DHI and DHICA (16), which are randomly linked via covalent bonds to form polymers. Its three-dimensional structure is strongly connected to the metal ions it binds with (24).

Selecting metal ions is a crucial step in designing nanomaterials for sustained drug release (25). These ions serve as a “structural scaffold” for the nanocarriers, shaping their architecture and stability, while also impacting drug loading, release behavior, and sometimes offering unique therapeutic effects (26). Among these factors, biocompatibility and toxicity are the most important considerations (27). Ca^2+^, Zn^2+^, and Mg^2+^, which are naturally present in the human body, generally show good biocompatibility and an acceptable safety profile (28). The EDS analysis revealed that the primary metal ion in GS eumelanin is Ca^2+^. Ca^2+^ mainly forms ionic bonds with carboxyl groups on the eumelanin polymer chains. This interaction is relatively weak and non-directional, primarily serving to “bridge” neighboring polymer chains and crosslink them through electrostatic attraction to create amorphous aggregates. These findings indicate that GS eumelanin is a pure, natural, and high-quality drug delivery vehicle.

XPS analysis confirmed that the purified GS eumelanin is primarily composed of carbon, oxygen, and nitrogen-elements that are fundamental to the DHI/DHICA polymerization structure of eumelanin (29). The detection of the N1s signal at ~399.8 eV is particularly significant, as it confirms the presence of indole-derived nitrogen species (pyrrole-type N and amide N), which are the structural hallmark of eumelanin (30). This finding provides direct evidence that the purified product retains the characteristic heterocyclic structure of eumelanin rather than being merely carbonized organic matter. The C1s deconvolution into C-C, C-O, and C=O peaks reveals a surface chemistry rich in both aromatic carbon and oxygen-containing functional groups, which is consistent with previous reports on natural eumelanin (29) and aligns with the FTIR observations (C=O at ~1,642 cm^−1^, C-O at ~1,115 cm^−1^).

The UV–Vis absorption spectrum of GS eumelanin exhibited the characteristic broad, featureless absorption profile typical of melanin pigments (29, 30). The gradual decrease in absorbance with increasing wavelength-a hallmark of melanin’s complex conjugated structure-is consistent with the characteristic negative slope observed in log absorbance versus wavelength plots for eumelanin (30). This spectral pattern confirms the retention of the DHI/DHICA backbone structure throughout the extraction and purification process. The absence of sharp absorption peaks in the visible region indicates the removal of low-molecular-weight chromophoric impurities, further corroborating the purity of the GS eumelanin preparation.

Multiple techniques (XPS, UV–Vis, SEM-EDS, and FTIR) characterized the surface chemistry, optical properties, and morphology of GS eumelanin. The material exhibits an irregular aggregated structure and a surface decorated with carboxyl, hydroxyl, and amino groups. Ca^2+^ likely coordinates with amino or carboxyl sites. The aromatic indole backbone remains intact, and the elemental composition falls within the typical range for eumelanin.

### 4.2 Detection of TXA and ACA in intestinal contents following GS eumelanin administration and their hemostatic mechanism

**Figure 8:**
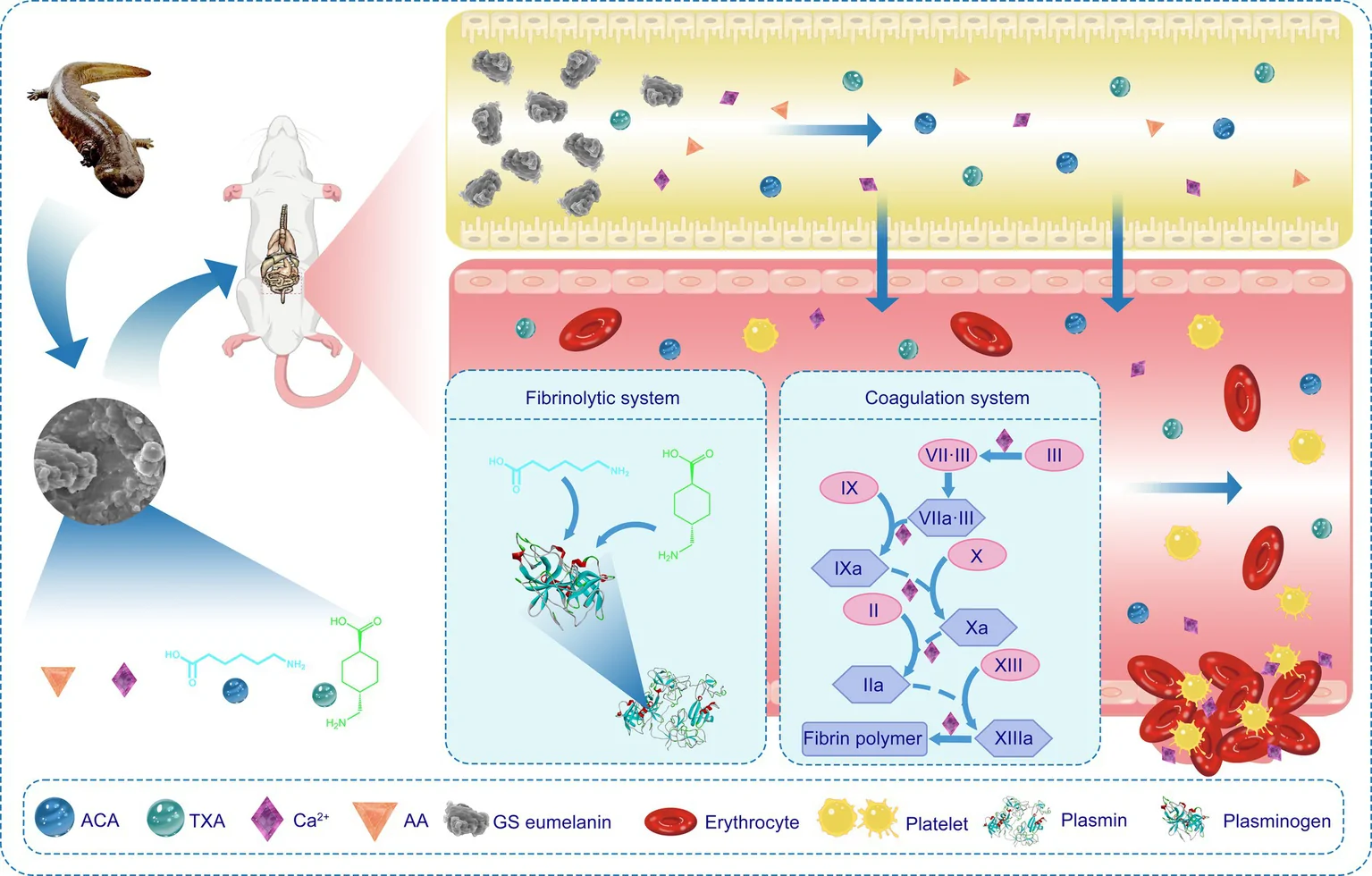
The mechanism by which GS eumelanin promotes hemostasis *in vivo*. GS eumelanin is metabolized in the intestinal tract into TXA and ACA, while also significantly increasing the levels of several amino acids and releasing Ca^2+^. TXA and ACA can bind to the plasminogen lysine site with high affinity, blocking plasminogen activation and inhibiting fibrinolysis. Ca^2+^, as a key cofactor in the coagulation cascade, speeds up the activation of coagulation factors and the formation of fibrin networks. The rat schematic image was created with BioGDP.com (45).

This study found that TXA and ACA were detected after GS eumelanin was metabolized in the intestine (Figure 8). Both TXA and ACA are lysine analogs with structures similar to those of lysine (31), enabling them to bind specifically to the lysine-binding sites on plasminogen. Through competitive binding, TXA and ACA effectively occupy these active sites, preventing plasminogen from binding to fibrin. This action prevents plasmin from forming and breaking down fibrin (32), thereby stabilizing blood clots and achieving effective hemostatic effects. Additionally, experiments demonstrated that low doses of TXA and ACA can effectively inhibit plasminogen activation. At higher concentrations, they can also directly inhibit plasmin’s proteolytic activity, further boosting hemostatic effectiveness (33, 34).

It is important to note that the detection of TXA and ACA in the intestinal contents following GS eumelanin administration, while they were not detected in the Con group, does not necessarily imply a direct biotransformation of eumelanin polymers into these compounds. There is a structural disparity between the DHI/DHICA monomers of eumelanin and the cyclohexane backbone of TXA and ACA. Therefore, the accumulation of these antifibrinolytic agents is unlikely to result from simple degradation of the polymer. Instead, it is more likely to reflect eumelanin-induced modulation of the intestinal microenvironment. This modulation may occur through changes in gut microbial composition or host metabolic pathways. TXA and ACA are lysine analogs. Their presence may also be linked to perturbations in lysine metabolism following GS eumelanin intervention. However, the definitive metabolic origin of these compounds remains unclear. Future studies should use stable isotope-labeled precursors, such as ^13^C-tyrosine, to trace the metabolic fate of GS eumelanin and its bioactive derivatives. These isotope tracing experiments will also help clarify whether TXA and ACA arise from lysine metabolism.

The *in vivo* metabolism of eumelanin is a complex process. It involves phase I and phase II metabolism, hydrolysis, oxidation, and sulfonation. These reactions work together. We used targeted metabolomics and standard compound comparison. We detected the presence of TXA and ACA. Based on this detection, we speculate that these two compounds may be at a downstream branch point of the metabolic pathway. However, we lack direct precursor-product enzyme kinetic data from the literature. Therefore, the complete transformation network is still unknown. The pathway we propose in this paper is only a tentative hypothesis.

### 4.3 Amino acid and Ca^2+^ mediate GS eumelanin procoagulant effects

This study found that after intestinal metabolism, GS eumelanin significantly increased the levels of several amino acids, including valine, threonine, isoleucine, aspartic acid, lysine, glutamate, histidine, phenylalanine, arginine, and tyrosine. Among these, lysine, aspartic acid, and arginine were especially elevated, along with the release of Ca^2+^. Most of these amino acids are essential and play key roles in coagulation. For example, lysine and arginine help activate coagulation factors VIII and X (35). Aspartic acid forms clusters of aspartic acid residues in the extracellular loop II of protease-activated receptor 4 (PAR4), which directly interacts with thrombin and acts as a critical site for platelet activation and aggregation (36). The overall increase in amino acid levels suggests that GS eumelanin may indirectly support coagulation homeostasis by enhancing amino acid availability, thereby promoting coagulation factor production, platelet function, and fibrin network stability. Furthermore, the release of Ca^2+^ strengthens this process (Figure 8), as Ca^2+^ is an obligatory cofactor for multiple coagulation reactions, including the activation of factors VII, IX, X, and XI, the stabilization of factor complexes on phospholipid membranes, and the formation of the prothrombinase complex-all of which are critical for efficient thrombin generation and fibrin clot formation (37, 38). This metabolic profile demonstrates that GS eumelanin directly positively modulates the coagulation system and manages the body’s amino acid balance and ion homeostasis through nutritional metabolic pathways, providing a new explanation for its multi-mechanistic synergistic effects in perioperative bleeding intervention.

### 4.4 The hemostatic efficacy and safety profile of GS eumelanin

Perioperative patients are often at risk of coagulation issues. Any hemostatic treatment must balance effectiveness with safety (39). This study evaluated the *in vivo* hemostatic activity of GS eumelanin. We used animal experiments. We compared our experimental values against established reference ranges. This comparison assessed whether the observed changes were within physiologically acceptable boundaries. One study established reference ranges for rats (40, 41). The ranges are PT 13.32–17.37 s, TT 16.08–58.20 s, APTT 10.54–29.90 s, and FIB 180–504 mg/dL. Another study reported similar SD values in rats at 10–20 weeks of age. That study reported PT 13.6 ± 2.7 s and APTT 17.2 ± 5.6 s.

At 2 h post-gavage, all parameters in the GS eumelanin groups fell within or close to the established ranges, supporting its safety profile and mitigating the risk of thrombosis. Specifically, the PT value in the H-GS group (15.8 ± 1.0 s) fell within the SD range (13.32–17.37 s), while the CON group (17.3 ± 1.1 s) slightly exceeded it, indicating that GS eumelanin normalizes PT rather than inducing pathological shortening-a hallmark of extrinsic pathway hypercoagulability. APTT values across all groups were within the accepted range of 10.54–29.90 s, with no group showing APTT shortening, directly arguing against intrinsic pathway hypercoagulability. TT values (32.3–35.1 s) aligned with published data and fell within the broad range of 16.08–58.20 s, excluding enhanced thrombin activity in the common pathway. FIB levels in the treated groups were higher than CON but remained under the upper limit; the CON value fell slightly below the lower limit (180 mg/dL) due to fasting, so the elevation in treated groups represents a restoration to baseline, not pathological hyperfibrinogenemia. Moreover, the maximum FIB value (230 mg/dL) is far below clinically meaningful thrombotic thresholds-human data indicate that FIB levels > 700 mg/dL are associated with significantly increased thrombotic risk. This safety profile is mechanistically plausible: the hemostatically active components derived from GS eumelanin are released gradually during digestion and metabolism, in contrast to clinical drugs such as tranexamic acid, which are rapidly absorbed upon intestinal entry. This slow-release characteristic avoids the abrupt high-dose antifibrinolytic exposure that may predispose to thrombotic complications. The concurrent pattern of PT normalization and FIB elevation toward baseline, with stable APTT and TT, further suggests that GS eumelanin acts selectively on the extrinsic and fibrinogen systems without globally activating the coagulation cascade (42). This profile fits a local hemostatic effect at the wound site rather than a systemic pro-thrombotic shift. In addition, although dose-dependent trends were observed for PT and FIB, the relatively narrow dose range (400–1,200 mg/kg) and small sample size precluded a robust dose–response assessment. Future studies with a wider dose range are needed to establish the therapeutic window. Coagulation parameters were measured only at 2 and 24 h post-gavage, which may not fully capture the temporal profile of the hemostatic effect; future studies with more frequent time points are needed.

Collectively, these findings show that GS eumelanin promotes hemostasis. It modulates coagulation parameters physiologically. It does not push any parameter outside safe boundaries or create a hypercoagulable state. Both reference range comparisons and clinically meaningful thresholds support this safety profile. Therefore, GS eumelanin has potential as a safe nutritional intervention for postoperative hemorrhage management.

Use of the tail-amputation model as a surrogate for postoperative hemorrhage. The tail-amputation bleeding model employed in this study is a well-established and widely used surrogate model for evaluating hemostatic efficacy in preclinical research (43). While it does not directly replicate the complex pathophysiology of surgical wound bleeding in humans, it offers several advantages: it provides a standardized and reproducible bleeding challenge with minimal inter-animal variability, allows precise quantification of bleeding time and blood loss, and is ethically preferable to more invasive surgical models. However, the translation of these findings to postoperative hemorrhage in humans requires further validation in clinically relevant surgical models. In addition, this study was conducted exclusively in male Wistar rats, which limits the generalizability of the findings to female subjects; future studies should include both sexes to assess potential sex-dependent effects.

An experiment on TXA in a rat model of polytrauma hemorrhage confirmed that TXA exerts early protective effects on coagulation (44). It does so mainly by transiently delaying PT prolongation. In our study, the GS eumelanin intervention directly shortened PT time. This is clearly different from the mode of TXA. TXA inhibits abnormal PT prolongation. GS eumelanin actively promotes the extrinsic coagulation pathway. TXA only counteracts coagulopathic prolongation caused by trauma. This difference suggests that the procoagulant effect of GS eumelanin may not be limited to preventing the breakdown of fibrin. It may also involve positive regulation of coagulation factors or contact activation.

### 4.5 Nutritional intervention and clinical translation of GS eumelanin

This study offers new insights for perioperative nutritional support. Traditional supplements include pure protein or amino acid mixtures. Unlike them, GS eumelanin is a bioactive dietary macromolecule. We preliminarily infer that, after gut metabolism, it exerts synergistic procoagulant effects through these three integrated pathways. These pathways include direct antifibrinolysis via TXA and ACA. They also provide essential amino acids. These amino acids serve as substrates for coagulation factors and platelet proteins. They also include the supply of calcium ions. Calcium ions are a key cofactor in the coagulation cascade. This structure–function integration moves GS eumelanin beyond mere nutrient supply. It makes it a natural dietary intervention. This intervention actively regulates coagulation homeostasis.

For clinical practice, GS eumelanin can be incorporated into postoperative dietary management in two ways. It can be added as a functional food ingredient to traditional soups or liquid diets. This leverages its natural origin and consumer acceptance. Alternatively, it can be developed into a standardized oral nutritional supplement (ONS). This ONS can be used for perioperative support in patients at high risk of postoperative bleeding. ONS is already widely recommended in enhanced recovery after surgery guidelines. The introduction of GS eumelanin could further expand the functional scope of ONS. It could expand from pure nutritional supplementation to active hemostatic regulation.

The doses used in this study (400–1,200 mg/kg in rats) were converted to human equivalent doses based on body surface area normalization. These calculations serve as a quantitative reference for dose translation rather than a direct recommendation for human intake. Notably, the present study employed a single acute administration model for efficacy evaluation, which differs fundamentally from chronic supplementation scenarios. The effective dose under realistic intake conditions-whether as a concentrated extract, capsule, or functional food ingredient-requires future optimization in humans. However, several practical factors support translational potential: (1) farm-raised giant salamanders provide a sustainable source of skin as a by-product; (2) GS eumelanin can be processed into a purified extract suitable for encapsulation or powder formulations; and (3) chronic use, if indicated, would likely require a lower maintenance dose than the acute regimen evaluated here. These considerations place the current findings within a feasible translational pathway, with human dose optimization as the logical next step.

Our findings indicate that GS eumelanin exhibits a potent hemostatic effect. However, further validation in additional models is required. This validation is needed before this agent can be translated into clinical nutritional interventions. Moreover, several key issues must be addressed, including its gastrointestinal fate, absorption kinetics, and systemic bioavailability-as eumelanin is generally poorly absorbed in its intact polymeric form-along with its long-term safety in specific populations and potential interactions with perioperative drugs such as anticoagulants and antiplatelet agents. Despite these limitations and unresolved questions, this study provides robust preclinical evidence for future translational research. It also highlights the potential importance of natural pigment-type dietary components. Eumelanin is one such component. These components matter in both nutritional science and coagulation medicine.

## 5 Conclusion

We successfully characterized eumelanin from the skin of farm-raised giant salamanders using SEM-EDS, FTIR, UV–Vis, and XPS, and examined its *in vivo* metabolites and hemostatic effects. The results show that GS eumelanin has a nanoscale aggregated structure with chelated Ca^2+^ within its framework and exhibits good biological safety. Following GS eumelanin administration, the antifibrinolytic metabolites TXA and ACA were identified in the intestinal metabolome. It also synergistically increases levels of several amino acids, including lysine and arginine, thereby creating a multi-target cooperative procoagulant mechanism. Animal experiments demonstrate that this mechanism significantly decreases hemorrhage volume and hemostasis time, while all coagulation parameters remain within safe ranges, indicating effective hemostatic safety. This study is the first to reveal the morphological structure, metabolic pathway, and hemostatic function of this eumelanin, providing a new approach for its use as a safe, effective, and mechanistically well-understood dietary intervention agent for managing postoperative hemorrhage.

## Data Availability

The original contributions presented in the study are included in the article/Supplementary material, further inquiries can be directed to the corresponding authors.
