# Supplementary material for: Eumelanin from the skin of farm-raised giant salamanders promotes coagulation system homeostasis: a nutritional intervention for postoperative hemorrhage
Source: Front Nutr. 2026 Aug 18;13:1896739. doi: 10.3389/fnut.2026.1896739 (PMC13529939; doi:10.3389/fnut.2026.1896739)
Supplement: Supplementary file 1 [file Table_1.DOCX]

Supplementary Material

# 1 Supplementary Tables

**Supplementary Table 1.** Optimal mass spectrometry parameters for TXA, ACA, and some major amino acids, including molecular ions, key sub-ions, and ion monitoring modes.

| No. | Compound ID | Metabolite identification | Formula | t_R_ (min) | Selected ion | Exact mass (Da) | Error (ppm) | Fragment ions | Related pathway |
| --- | --- | --- | --- | --- | --- | --- | --- | --- | --- |
| 1 | HMDB0014447 | TXA | C8H15NO2 | 9.98 | [M+H]+ | 158.11756 | 0.758929 | 95.08603; 141.15106; 123.08055 |  |
|  |  |  |  |  | [M-H]- | 156.10300 | 0.576542 | 57.97446;85.00301; 89.02306 |  |
| 2 | HMDB0001901 | ACA | C6H13NO2 | 9.83 | [M+H]+ | 132.10191 | 1.059788 | 114.09141;69.07043; 96.08105 |  |
|  |  |  |  |  | [M-H]- | 130.08735 | 1.229943 | 61.98692;102.98723; 111.04371 |  |
| 3 | HMDB0000883 | L-Val | C5H11NO2 | 6.69 | [M+H]+ | 118.08626 | 2.709883 | 72.08142; 57.05801; 55.05497 | Val, Leu and Ile biosynthesis |
|  |  |  |  |  | [M-H]- | 116.07170 | 2.239995 | 71.01250; 99.92423; 89.01333 |  |
| 4 | HMDB0000167 | L-Thr | C4H9NO3 | 7.17 | [M+H]+ | 120.06552 | 2.415348 | 74.06068;102.05531; 56.05024 | Val, Leu and Ile biosynthesis |
|  |  |  |  |  | [M-H]- | 118.05097 | 1.524765 | 74.02335; 72.00770; 109.54774 |  |
| 5 | HMDB0000172 | L-Ile | C6H13NO2 | 6.13 | [M+H]+ | 132.10191 | 0.963355 | 86.09694; 69.07048; 61.01134 | Val, Leu and Ile biosynthesis |
|  |  |  |  |  | [M-H]- | 130.08735 | 1.036132 | 95.02364; 114.01823; 70.02843 |  |
| 6 | HMDB0000191 | L-Asp | C4H7NO4 | 8.21 | [M+H]+ | 134.04478 | 3.940179 | 87.02685; 133.03189; 74.02430 | Arg biosynthesis; His metabolism |
|  |  |  |  |  | [M-H]- | 132.03023 | 4.9681 | 88.03896; 115.00226; 71.01247 |  |
| 7 | HMDB0000182 | L-Lys | C6H14N2O2 | 9.74 | [M+H]+ | 147.11280 | 3.670653 | 84.04494；102.05531；130.04993 | Biotin metabolism |
|  |  |  |  |  | [M-H]- | 145.09825 | 0.089594 | 102.05463；128.03397； 129.47148 |  |
| 8 | HMDB0060475 | L-Glu | C5H9NO4 | 8.09 | [M+H]+ | 148.06043 | 0.384444 | 84.04491; 102.05453; 130.04990 | Arg biosynthesis; His metabolism |
|  |  |  |  |  | [M-H]- | 146.04588 | 4.276195 | 102.05469; 128.03400; 61.98703 |  |
| 9 | HMDB0000177 | L-His | C6H9N3O2 | 9.23 | [M+H]+ | 156.07675 | 0.222087 | 110.07158; 95.06078; 83.06092 | His metabolism |
|  |  |  |  |  | [M-H]- | 154.06220 | 2.1581 | 137.03429; 93.04441; 72.00768 |  |
| 10 | HMDB0000159 | L-Phe | C9H11NO2 | 5.95 | [M+H]+ | 166.08626 | 1.508724 | 120.08094；103.05449；131.04910 | Phe, Tyr and Try biosynthesis |
|  |  |  |  |  | [M-H]- | 164.07170 | 1.730182 | 119.04889；147.04382；72.00771 |  |
| 11 | HMDB0000517 | L-Arg | C6H14N4O2 | 9.56 | [M+H]+ | 175.11895 | 0.731361 | 158.08116; 129.10231; 84.04491 | Arg biosynthesis |
|  |  |  |  |  | [M-H]- | 173.10440 | 4.727141 | 155.08128; 128.03397; 84.04409 |  |
| 12 | HMDB0000158 | L-Tyr | C9H11NO3 | 6.28 | [M+H]+ | 182.08117 | 4.769045 | 136.07568; 165.05458; 123.04420 | Phe, Tyr and Try biosynthesis |
|  |  |  |  |  | [M-H]- | 180.06662 | 4.535414 | 163.03879; 119.04886; 93.03321 |  |
| 13 | HMDB0000161 | L-Ala | C3H7NO2 | 6.83 | [M+H]+ | 90.05495 | 3.953903 | 62.17123;72.04501; 87.00449 | Ala, Asp and Glu metabolism |
|  |  |  |  |  | [M-H]- | 88.04040 | 3.484051 | 60.99170;71.01242; 59.01243 |  |
| 14 | HMDB0000187 | L-Ser | C3H7NO3 | 7.73 | [M+H]+ | 106.04987 | 1.55342 | 60.04514; 88.03979; 70.02937 | Glyoxylate and dicarboxylate metabolism |
|  |  |  |  |  | [M-H]- | 104.03532 | 3.697468 | 87.92390;72.00771; 74.02335 |  |
| 15 | HMDB0000929 | L-Try | C11H12N2O2 | 6.94 | [M+H]+ | 205.09715 | 4.036476 | 120.08084; 166.08607; 188.07047 | Try metabolism |
|  |  |  |  |  | [M-H]- | 203.08260 | 1.882357 | 164.07048; 132.02892;61.98700 |  |

**Supplementary Table 2.** Targeted metabolomic analysis of intestinal amino acids in CON and H-GS eumelanin rats.

| No. | Metabolite identification | Formula | CON (μM) | H-ECGS (μM) |
| --- | --- | --- | --- | --- |
| 1 | TXA | C8H15NO2 | / | 828.82±73.07 |
| 2 | ACA | C6H13NO2 | / | 2199.50±174.72 |
| 3 | L-Val | C5H11NO2 | 18658.53±2116.87 | 25371.09±2820.12 |
| 4 | L-Thr | C4H9NO3 | 1572.15±130.93 | 3064.62±152.37 |
| 5 | L-Ile | C6H13NO2 | 6065.12±478.51 | 9106.25±1323.65 |
| 6 | L-Asp | C4H7NO4 | 1143.54±305.65 | 3696.37±842.48 |
| 7 | L-Lys | C6H14N2O2 | 3141.87±1058.75 | 5151.78±465.26 |
| 8 | L-Glu | C5H9NO4 | 5513.54±248.63 | 7767.22±146.15 |
| 9 | L-His | C6H9N3O2 | 1622.63±388.90 | 2618.01±350.40 |
| 10 | L-Phe | C9H11NO2 | 223.96±75.51 | 2446.99±865.10 |
| 11 | L-Arg | C6H14N4O2 | 1742.11±54.32 | 3694.59±83.15 |
| 12 | L-Tyr | C9H11NO3 | 1008.41±147.42 | 1923.37±306.19 |
| 13 | L-Ala | C3H7NO2 | 2570.25±731.83 | 2611.90±1259.98 |
| 14 | L-Ser | C3H7NO3 | 681.64±81.20 | 410.93±164.51 |
| 15 | L-Try | C11H12N2O2 | 794.45±81.09 | 785.91±33.59 |

**Supplementary Table 3.** Extraction recoveries of TXA and ACA from the H-GS eumelanin rats’ intestinal matrix (n = 3).

| No. | Spike compound | Sample amount (µg) | Spike amount (µg) | Recovery (%) |
| --- | --- | --- | --- | --- |
| 1 | TXA | 65.1448 | 30.43 | 95.94±3.27 |
| 2 | ACA | 144.254 | 71.39 | 94.64±2.97 |
